# Digital Leisure Engagement and Positive Outcomes in the Workplace: A Systematic Literature Review

**DOI:** 10.3390/ijerph20021014

**Published:** 2023-01-05

**Authors:** Joschka Gellmers, Nanxi Yan

**Affiliations:** 1 Department of Psychology, Faculty of Behavioural and Social Sciences, University of Groningen, 9712 CP Groningen, The Netherlands; 2 Department of HRM & OB, Faculty of Economics and Business, University of Groningen, 9747 AJ Groningen, The Netherlands

**Keywords:** digital leisure, employee recovery, employee productivity, employee well-being, cyberloafing, systematic review, work and leisure

## Abstract

The rise of the Internet and information and communication technologies (ICTs) has led to employees spending increasingly more time on non-work-related digital activities at work. A vast literature base exists that is devoted to the potential adverse effect of such activities in the form of cyberloafing. However, not much is known about the positive outcomes of such activities conceptualized as digital leisure. The present review systematically examines current literature on digital leisure activities and how these contribute to positive outcomes in the workplace. Additionally, possible moderating and mediating variables are investigated. Using the preferred reporting items for systematic reviews and meta-analyses (PRISMA) framework, eight peer-reviewed studies were identified that met inclusion criteria. The results indicate that resource recovery processes and employee well-being, as well as employee productivity are positively associated with digital leisure in the workplace. Age was found to moderate the relationship between digital leisure and self-reported employee productivity, while employee satisfaction was found to mediate the relationship between digital leisure and employee productivity. Future research directions are outlined and implications for the work context are discussed.

## 1. Introduction

The emergence and advancement of information and communication technologies (ICTs) has revolutionized the workplace, that facilitates employees’ access to different work platforms and resources [1], but has also changed leisure practices [2] and where and when leisure takes place [3,4]. Historically, work and leisure were separated life domains [5], neatly divided by time and space. However, nowadays, the possibility of almost constant mobile connectivity [6] opens up opportunities to engage in ICT-based leisure activities in the workspace and during working time [4]. This blurring of work–leisure boundaries [7] has led to two streams of literature that approach such digital activities from different theoretical perspectives. First, a large research body based in the organizational sciences conceptualizes these digital activities as cyberloafing; defined as the non-work-related personal use of an organization’s internet [8]; these activities are considered to be undesirable counterproductive work behaviors [9]. However, at the same time, as people started spending increasingly more time on digital activities in the workplace [10], a second stream of literature emerged, based in the field of leisure studies. Scholars in this field of digital leisure studies conceptualize ICT-based leisure activities as digital leisure [4,6,10] and explore the meaning and potential positive outcomes of such leisure activities (e.g., social media use, mobile computer games) at work.

While previous research has often adopted a cyberloafing perspective and heavily focused on aspects of counterproductive behavior [11], this systematic literature review aims to shed light on the neglected positive benefits of digital activities, specifically on (a) how and why non-work-related digital activities contribute to positive outcomes in the workplace and (b) investigate which circumstances make such positive outcomes more or less likely. By organizing and integrating the current state of research on digital activities in the workplace, this review makes the following contributions. First, we distinguish digital leisure from cyberloafing based on current research in organizational research and leisure studies to emphasizes its unique recovery aspect warranting more scholarly attention. Next, we systematically reviewed extant literature across fields of study and identified the potential recovery benefits of digital activities. Performing a cross-disciplinary review provides both organizational scholars and leisure researchers with a more balanced and complete understanding (e.g., different ways) of digital activities in the workplace which cross-fertilizes each field of study and drives future research. Furthermore, our review provides practitioners with evidence-based knowledge to apply at both an organizational (e.g., work design) and individual level (e.g., self-management strategies).

### Theoretical Background

While both cyberloafing and digital leisure can essentially describe any—if not the same—digital activity at work that is not work-related, it is important to note that digital leisure is distinct from cyberloafing. First, digital leisure is characterized by a subjective experiential component. Traditionally, leisure is defined by the core aspects of action, time and experience [12]. Action refers to an activity that individuals choose to engage in [3], while the time dimension is the residual aspect of leisure and refers to leisure as time that is not occupied by work [13] or other obligations and duties (such as household chores, sleep, etc.). Lastly, experience forms the subjective component of leisure [12]. This component takes into consideration that an action, as well as time, have to be perceived and construed as leisure. Importantly, while digital leisure clearly challenges the traditional divide between work- and leisure-spaces and work and leisure time, such activities can very much be subjectively experienced as leisure. In other words, it is not necessarily action or time, but the experience of it that characterizes digital leisure [4]. Cyberloafing, in contrast, does not take into account how an activity is experienced but instead looks at the activity and time aspect (e.g., engaging in social media consumption during working time). As such, a digital leisure perspective adds an important component to the understanding of digital activities in the workplace as it takes the individual employee perspective into consideration.

Second, research on cyberloafing predominantly focuses on the adverse effects of these digital activities [11], such as a decrease in employee productivity (e.g., [14]). A digital leisure perspective, on the other hand, shifts the research focus towards the positive outcomes of digital activities at work. There is ample research showing that leisure activities can have various beneficial outcomes in the work context. Particularly, leisure can function as a source of recovery from work stressors and job demands (e.g., [15,16]) and, as such, leisure is important for overall well-being at work [13,17] as well as work performance (e.g., [18]).

In the workplace, according to the job demands–resources model (JD-R, [19]), employees are exposed to job demands (e.g., work overload) that require physical or mental effort. These demands can, eventually, deplete employee resources. Similarly, the effort–recovery model [20] states that employee resources are depleted as employees expend effort and make use of resources to accomplish their work tasks. In contrast, employee resources include organizational, social and physical aspects in the workplace that can aid in a reduction of job demands [19] and internal psychological resources, such as motivation, concentration or mental energy [21]. These internal resources are limited and, thus, can be depleted by work stressors and job demands. In order to prevent work stress and subsequent negative well-being and health outcomes [15], these depleted internal resources need to be replenished, a process called resource recovery [22]. There is evidence that digital leisure activities can function as a source for resource recovery [23] and that such activities can restore attentional resources in the workplace [24], help with workplace recovery [23] and improve employee performance [25].

The replenishment or recovery of these psychological resources by means of leisure activities can take different forms. Sonnentag and Fritz [16] distinguish between four types of recovery experiences. First, individuals can detach from a current work task, this can be either psychologically or physically. Second, individuals can engage in relaxation which, essentially, is a state of low psychophysiological activation and as such can restore positive affect. Third, mastery experiences may help the individual replenish their mental resources as they can, resulting, for example, in the experience of competence. Lastly, although not a ‘recovery strategy’ per se, autonomy or a sense of control plays a crucial role for the experience of leisure [26]. Leisure is characterized by a high degree of freedom or choice [26] and perceiving an activity as freely chosen is, thus, essential. Digital leisure has been linked to inducing resource recovery by eliciting all four recovery experiences [27]. Similar to the findings by Sonnentag and Fritz [16], Newman and Diener [17] identified detachment, mastery experiences and control as central psychological mechanisms that are triggered by leisure activities, which can subsequently lead to an increase in subjective well-being. Additionally, they found that meaning and social affiliation may function as such mechanisms; leisure that is personally meaningful (i.e., relevant) and takes place in a social context (i.e., fulfills affiliate needs) can thus increase subjective well-being.

In sum, approaching non-work-related digital activities in the workplace from a digital leisure perspective shifts the research focus towards the potential positive outcomes of these activities. Findings suggest that digital leisure in the workplace, similar to other forms of leisure activities, can elicit recovery processes and, hence, improve employee well-being and performance. Despite the potential of digital leisure for recovery and employee well-being and the important consequences this might have for a more holistic, interdisciplinary understanding of non-work-related activities in the workplace, currently—to our best knowledge—no attempt has been made to summarize research findings on digital leisure in the workplace.

Therefore, the current review aims to address this gap in research by (a) organizing and synthesizing the current state of knowledge regarding digital leisure activities in the workplace to gain a better understanding of how digital leisure contributes to positive outcomes in the workplace. Furthermore, (b) to further understand circumstances under which digital leisure engagement is most and least likely to benefit employees, possible moderators and mediators are investigated. For example, as described earlier, perceived control plays an important role in the experience of leisure [3,16]. If, say, company policy states that any form of digital activities that are not work-related are prohibited, this might lead to such activities not taking place. If employees cannot freely decide to engage in digital leisure as a source of resource replenishment, no positive benefits can be expected from it. Another possible moderating variable might be age. As the workforce is changing, and younger workers enter the workforce [7], employees that grew up with digital leisure might be more inclined to engage in such activities in the workplace.

In the following, this review is organized in six sections. First, the methodological approach of this systematic literature review is described. Second, the results are presented, followed by, third, a discussion of the reviewed literature. Fourth, recommendations for future research directions are outlined. Fifth, practical implications for the work context are given. Lastly, the limitations of this review are critically discussed.

## 2. Method

This systematic literature review followed the preferred reporting items for systematic reviews and meta-analyses (PRISMA) guidelines [28] for a structured reporting of identified, screened, deemed eligible and included studies. PRISMA guidelines consists of a 27-item checklist to ensure the transparency and thoroughness of the review process [28]. Additionally, for the process of data extraction the steps and procedures recommended and summarized by Boland et al. [29] were followed. The protocol of this systematic review was registered in OSF (https://osf.io/5jgyx/, accessed on 1 January 2023), and this review followed the protocol.

### 2.1. Search Strategy and Screening Process

**Figure 1:**
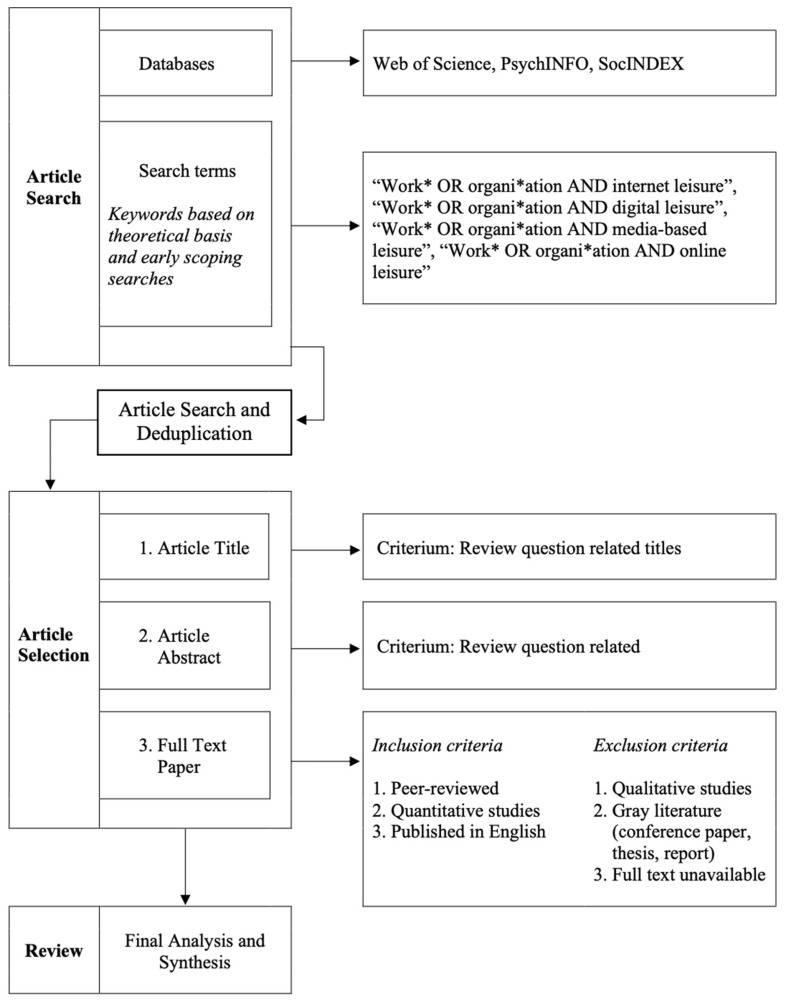
Overview of article search, article screening and the article selection process.

Several scoping searches preceded the actual literature review. Scoping searches are preliminary literature searches that are conducted to gauge if a certain topic is suitable for a systematic review and the possible scale of it [29]. In addition, such searches provide an overview of the existing literature and help to identify possible background literature [29]. Lastly, as part of the scoping search PROSPERO was searched. Furthermore, for the review to have a broad interdisciplinary scope, the electronic databases Web of Science, PsychINFO and SocINDEX were searched using Boolean search strings (Figure 1). It was decided to limit the search to literature published from 2005 to 2022 (including the years 2005 and 2022). This search period was chosen because digital leisure activities in the workplace became increasingly more prevalent and likely with the rise of, e.g., social media and online video platforms [30]. These changes took place, approximately, between the year 2000 and 2010 with the emergence of the so-called Web 2.0 [30]. For the review, three literature searches were conducted on 21 April, 22 April, and 23 April 2022. Once the final papers were selected and obtained, the reference lists of those papers were searched for additional citations.

**Figure 2:**
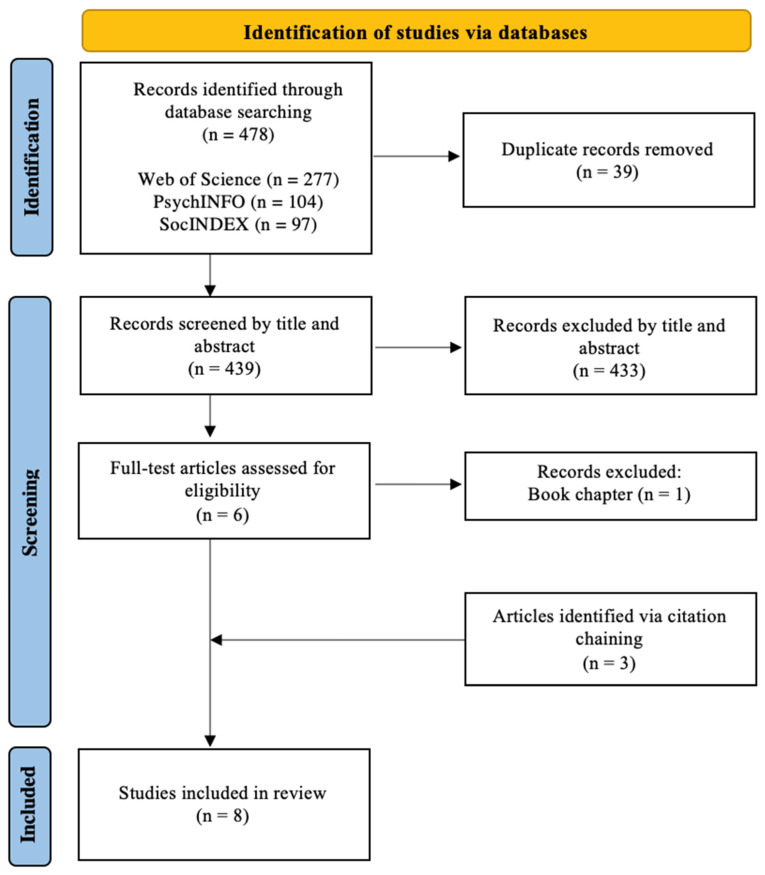
Article search, article screening and article selection.

Furthermore, electronic databases were used for cross-reference searches to locate additional relevant literature. The primary author conducted the search and literature collection. Although the primary author conducted the screening independently, the inclusion and exclusion criteria was established in consultation with the second author. After the citations were exported and the duplicates were removed, the primary author examined titles and abstracts to determine inclusion eligibility. If the relevance of an article could not be determined through the title or abstract, the primary author would investigate the full text of the article. Both authors met regularly to discuss the disputes detected during the screening process and achieve consensus on disparities. Endnote (version 2.0) was used to manage the obtained citations and identify possible duplicates in the literature (see Figure 1 for the full review process). Overall, electronic searches resulted in 478 citations of which eight articles met eligibility requirements (see Figure 2).

### 2.2. Inclusion and Exclusion Criteria

Before conducting the systematic review, inclusion and exclusion criteria were established. This review focused on reviewing peer-reviewed articles in order to ensure the quality of publications. Furthermore, studies were eligible if they were quantitative and published in English. Conference papers, theses and reports and other gray literature, as well as qualitative studies, were excluded from this review. Lastly, studies were also excluded if no full-text version was available. The inclusion and exclusion criteria were chosen due to theoretical relevance and prior scoping searches, without an underlying a priori framework.

As recommended by Boland et al. [29], the inclusion and exclusion criteria were revisited and reevaluated as during the process of becoming more familiar with the relevant literature. As a particular consequence, as earlier searches yielded a very small sample of citations, it was decided to adapt the exclusion criteria and include studies that made use of student samples.

## 3. Results

The eight studies included in the final review are presented in Table 1. Five studies were cross-sectional, two made use of an experimental design and one study used cross-sectional and experimental data. These studies were published between 2009 and 2019 and conducted in various countries; one in the USA, China, Malaysia, Germany, and South Korea and three in Australia. The majority of studies (75%) used employees as a sample, while one study used both students and employees. Of the employee samples, three samples consisted of not further specified full-time workers, two samples were composed of office workers, one sample consisted of bank employees, and one sample of telecommunication service employees. The sample size ranged from 127 to 1208 participants (see Table 2 for the full data extraction).

Of the reviewed articles, seven were theoretically grounded in resource and recovery theories. As such, it was argued that digital leisure activities in the workplace can provide the employee with resources (see job demands–resource model, [19]), prevent the depletion of employee resources (see conservation of resources theory, [31]; ego depletion theory, [32]) and improve recovery and replenishment of resources once these are depleted (see Recovery Experience Questionnaire, [16]). Three articles would additionally base theoretical assumptions in self-determination theory (SDT, [33]) and emphasize the motivational components control (or autonomy) and relatedness, while one article would investigate the social–organizational aspect of digital leisure activities and focus on the theoretical perspective of social penetration theory [34].

**Table 1:** Summary of Reviewed Articles.

Author(s)		Year	Country	Journal
1.	Coker [35]	2011	Australia	*New Technology*
2.	Coker [24]	2013	Australia	*Human Performance*
3.	Janicke et al. [36]	2018	Australia	*Mass Communication and Society*
4.	Janicke-Bowles et al. [37]	2019	USA	*Journal of Happiness Studies*
5.	Luo et al. [38]	2017	China	*Computers in Human Behavior*
6.	Mohammad et al. [25]	2019	Malaysia	*Internet Research*
7.	Reinecke [39]	2009b	Germany	*CyberPsychology & Behavior*
8.	Rhee and Kim [40]	2016	South Korea	*Computers in Human Behavior*

**Table 2:** Data Extraction.

Study	Sample (Age Range, *M*_Age_, *SD*_Age_)	Type Sample (1 = Worker, 2 = Student)	Design	Focus	Leisure Source	Main Findings
Coker (2011) [35]	268 office workers, 74% female (M = 33)	1	Cross-sectional	Workplace productivity	General Internet use	1. Individuals that engage in workplace Internet leisure browsing (WILB) had higher self-reported workplace productivity scores than those who do not/cannot engage in WILB. 2. The relationship between WILB and productivity was curvilinear. WILB can have a positive effect on worker productivity if percentage of WILB does not exceed more than 12 per cent of work time. 3. WILB breaks showed a positive relationship against worker performance when duration and overall amount of time spent WILBing was controlled for.
Coker (2013) [24]	Study 1: 127 university business school students, 50% female (M = 21) Study 2: Sample overlap with [1], but additional findings	1, 2	Experiment (Study 1) Cross-sectional (Study 2)	Task vigilance Work productivity	Social media (Facebook)	1. WILB replenishes attentional resources and by doing so increases vigilance. 2. Study 1: Enjoyability of breaks moderates the restoration of attentional restoration/WILB replenishes attentional resources more than less enjoyable types of breaks. 3. Study 2: Workplace Internet leisure browsing predicted perceived productivity for individuals brought up with the Internet (those younger than 30).
Janicke et al. (2018) [36]	148 full-time employees, female 47% (M = 36.26, SD = 10.72)	1	Experiment (online)	Workplace well-being	YouTube videos	1. Meaningful videos predicted mastery recovery experiences. 2. Positive affect predicted psychological detachment and relaxation experiences. 3. Mastery recovery experiences predicted vitality, whereas relaxation experiences predicted work satisfaction.
Janicke-Bowles et al. (2019) [37]	200 full-time workers, 48% females, 51.5% males and 0.5% transgendered (20–68, M = 37.11, SD = 11.05)	1	Experiment (online)	Work-related well-being	YouTube videos	1. Short eudaimonic entertainment experiences (with meaningful/inspiring content) as elicited from a 3–5 min YouTube video at work can impact short-term psychological well-being; subjective well-being (operationalized as vitality), psychological well-being (operationalized as meaning at work), and social well-being (operationalized as relatedness at work). 2. Any form of media consumed over a period of 3–4 min reduced employees’ stress levels.
Luo et al. (2017) [38]	1208 mobile telecommunications service provider workers, 58.11% female	1	Cross-sectional	Affective organizational commitment	Social media (corporate blog)	1. Non- work-related content contribution and information acquisition activities by means of blogging correlated positively with affective organizational commitment of employees.
Mohammad et al. (2019)	282 bank employees, female 56.4%	1	Cross-sectional	Employee satisfaction and productivity	General Internet use	1. Workplace Internet leisure (WIL) had a positive effect on employees’ satisfaction. 2. Employee satisfaction mediated the relationship between WIL and employee productivity.
Reinecke (2009b) [39]	833 German users of game portal that are employed, female 53.5% (16–66, M = 35.1, SD = 9.9)	1	Cross-sectional	Workplace recovery (experience)	Computer games	1. Playing computer games in the workplace elicits substantial levels of recovery experience. 2. Individuals with higher levels of work-related fatigue reported stronger recovery experience during gameplay and showed a greater tendency to play games during working hours than did persons with lower levels of work strain. 3. Social situation at work had a significant influence on use of games: individuals receiving less social support from colleagues and supervisors played games at work more frequently than did individuals with higher levels of social support to cope with work related fatigue.
Rhee & Kim (2016) [40]	425 office workers, 35% female (M = 39.05, SD = 7.99)	1	Cross-sectional	Workplace vitality	Smartphone use	1. Psychological detachments by breaks, independent of break modes, led to increase in vigor and reduce emotional exhaustion. 2. However, effects, particularly in reducing emotional exhaustion, were significantly lower for the smart phone break group versus the conventional groups.

### 3.1. Positive Outcomes of Digital Leisure at Work

The literature search revealed that studies on digital leisure activities in the workplace fall into two main categories: employee recovery and well-being (five articles) and employee productivity (three articles) and organizational commitment (one article).

#### 3.1.1. Employee Recovery and Well-Being

Two studies [36,37] were identified that looked at the effects of watching online videos on recovery and well-being outcomes. An online experiment [36] with 148 full-time employees investigated the role of video content on recovery experiences and work satisfaction in the workplace. The researchers had participants watch short YouTube videos. These videos were—depending on the content shown—expected to either elicit funny (hedonic) or meaningful (eudaimonic) experiences, compared to neutral videos that were presented to the control group. They found that videos with funny (hedonic) content predicted positive affect in participants, which in turn would predict the recovery experiences: psychological detachment and relaxation. Furthermore, videos that elicited a sense of meaningful (eudaimonic) experience in the form of gratitude and elevation predicted mastery experiences. These, in turn, predicted participants’ work satisfaction. Comparable effects were found in another online experiment [37] among 200 full-time employees. The researchers looked at the effect of hedonic and eudaimonic short-form YouTube videos on subjective well-being, psychological well-being and social well-being at the workplace. The researchers operationalized subjective well-being as vitality (an increase indicating a form of stress reduction), psychological well-being as meaningfulness at work, and social well-being as a sense of relatedness at work. Significant positive effects were found for subjective well-being, psychological well-being, and social well-being when participants watched eudaimonic short-form YouTube videos. It should be noted that any form of short-form media consumption was positively associated with subjective well-being (vitality in the form of stress reduction)—whether participants watched videos containing hedonic or eudaimonic content, or a neutral slideshow (control condition).

One study [40] investigated the effects of psychological detachment from work in the form of smartphone micro-breaks and conventional breaks (defined as no engagement with electronic devices) on vigor and emotional exhaustion among 425 surveyed office workers. Vigor was defined as high levels of energy and mental resilience, while emotional exhaustion was defined as the degree to which one feels drained of emotional energy and fatigued. The authors hypothesized that psychological detachment would be positively associated with vigor by increasing positive affect and negatively associated with emotional exhaustion by decreasing negative affect. They found that smartphone breaks were indeed positively related with an increase in vigor and a reduction in emotional exhaustion. However, it should be noted that a significant difference was found between smartphone breaks and conventional breaks for the reduction of emotional exhaustion. In comparison, conventional breaks (i.e., taking a walk) were better at reducing emotional exhaustion.

Another study [39] looked specifically at the relationship between computer games in the workplace and recovery experiences (see [16]) in a large surveyed sample of 833 employed game portal users. The author found that playing computer games at work was positively associated with psychological detachment, relaxation, mastery experiences, and control. Furthermore, exhaustion in the form of work-related fatigue and having a sense of job control were positively related to actually engaging in computer games in the workplace. In other words, exhausted workers and workers that perceived being able to engage in computer games were more likely to do so. Lastly, reported levels of social support in the working environment were negatively related to engaging in computer games, such that participants that reported lower levels of social support made more use of computer games as a source of recovery experience.

#### 3.1.2. Workplace Productivity

Three studies [24,25,35] were identified that explored the potential relationship of digital leisure activities and workplace productivity (for Mohammad et al. [25], see section Moderators and Mediators). Coker [35] investigated the relationship of digital leisure in the form of workplace internet leisure browsing (WILB) on workplace productivity in an online survey among 268 office workers. The study found that higher self-reported WILB activity scores were positively associated with higher self-reported productivity scores. Employees that engaged in WILB had 9 percent higher workplace productivity scores than employees that could not (or did not) engage in WILB. Furthermore, it was found that shorter, more frequent periods of WILB showed a greater positive effect on productivity than longer but less frequent periods of WILB. However, it should be noted that the relationship between WILB and employee productivity appeared to be curvilinear. While WILB, initially, was positively related to employee productivity, this effect diminished when more than 12 percent of working time was spent on WILB. The authors suggest that WILB might work as a micro-break that restores concentration and requires a certain degree of job control for it to take place. However, no measures of, e.g., resource recovery or replenishment or job control were added to the survey, hence the explanatory mechanism remained speculative.

In a follow-up experiment, Coker [24] investigated the effect of WILB-breaks in the form of Facebook engagement on task vigilance. For this, 127 university business school students performed a vigilance task. The students were assigned to four groups: a control group, a non-active group, an Internet task group, and a WILB group. The control group did not take any break during the vigilance task, while the non-active group was instructed to simply remain seated while taking their break. The Internet task group had to perform a high cognitive load task in which participants compared health insurances during the break. Lastly, the WILB group was allowed to browse their Facebook during their break. The experiment found evidence that WILB-breaks resulted in a significantly greater sustained task vigilance in comparison to the other experimental conditions. The author argued that a more enjoyable task would lead to the replenishment of attentional resources which prevented a decay of task vigilance.

#### 3.1.3. Organizational Commitment

One study [38] investigated digital leisure from a social networking perspective in a large sample of 1208 employees. The researchers were interested in the relationship between corporate blogging engagement in the form of writing and reading non-work-related blog posts and affective organizational commitment. It was hypothesized that contribution to a corporate blog was a form of self-disclosure (sharing information about oneself) and reading others’ non-work-related content would help to establish shared understanding among coworkers. Both content contribution and acquisition (reading coworkers’ content), in turn, would increase affective organizational commitment. The hypotheses were supported; both content contribution and acquisition were positively associated with affective organizational commitment.

### 3.2. Moderators and Mediators

Two studies were identified that investigated possible moderators or mediators. One of the aforementioned studies [24] found employee age as a possible moderator for the relationship of digital leisure on employee productivity and one study [25] looked into the mediating role of employee satisfaction in the relationship of digital leisure on employee productivity.

#### 3.2.1. Employee Age as a Moderator

Coker [24] looked at the data of a sample of 268 office workers (note the sample overlap with Coker [35]). The author found that employee age moderated the relationship between workplace internet leisure browsing (WILB) and perceived workplace productivity, such that only younger workers (≤30 years), brought up with the internet, perceived productivity benefits, while older workers did not. The author suggests that younger employees might be more likely to perceive WILB as actual leisure and, thus, benefit from such behavior.

#### 3.2.2. Employee Satisfaction as a Mediator

Mohammad et al. [25] surveyed 282 bank employees and looked at the relationship of workplace internet leisure (WIL) and employee productivity and found that employee job satisfaction mediated this pathway. More specifically, the authors argue that workplace internet leisure (WIL), workplace autonomy orientation (WAO), and workplace internet leisure policy (WILP) are employee resources that predict employee job satisfaction, which in turn predicts employee productivity. According to the authors, WIL provides a form of break, while WAO provides employees with the necessary perceived job control (i.e., freedom) to engage in WIL. Lastly, and related to WAO, a company policy that clearly states, and fairly (i.e., permits such behavior to a certain degree) regulates, non-work-related digital activities in the workplace can improve employee satisfaction.

## 4. Discussion

Over the last few decades, technology advancements, decentralized work arrangements, and the growing interests in the promotion of recreation activities in the workplace vanished the boundaries between work and leisure which made digital leisure engagement a prominent phenomenon in the workplace. Current literature on this topic either focused on cyberloafing or workplace recreation activities in general, while fewer studies examined digital leisure engagement. The current literature review aimed to contribute to a more complete, interdisciplinary understanding of non-work-related digital activities at work by systematically organizing and synthesizing literature on how digital leisure can contribute to positive outcomes in the workplace. In addition, to understand the circumstances under which digital leisure engagement is most and least likely to benefit employees, possible mediators and moderators were examined. Eight studies were identified that investigated positive outcomes of digital leisure in the workplace. The findings of this review suggest that studies on digital leisure activities fall into two main categories of employee recovery and well-being and workplace productivity and affective organizational commitment.

The first set of studies looked at the role of digital leisure in the process of employee resources recovery and well-being. Findings from this review suggest that digital leisure activities in the workplace can contribute to employee resource recovery processes and employee well-being. More specifically, digital leisure in the form of short videos appeared to be associated with employee recovery in the form of psychological detachment and relaxation [36]. Furthermore, playing computer games in the workplace can foster recovery by means of detachment, relaxation, mastery experiences and was positively associated with perceived control [39]. Short-form videos were also associated with the recovery experience of mastery experiences and, subsequently, positively related with an increase in satisfaction with work [36]. In addition, watching short online videos was related to outcomes of subjective well-being (vitality), psychological well-being (meaningfulness at work), and social well-being (relatedness) [37]. Adding to this, review findings suggest that digital leisure can be a social activity and as such fulfill affiliation needs [17]. Interestingly, the results of one reviewed study [39] suggest that digital leisure in the form of computer games might contribute to the reduction of work stress when no social support at work is available [39]. Lastly, smartphone breaks were related to psychological detachment, an increase in vigor and a reduction in emotional exhaustion [40].

The second set of studies focused on digital leisure at work and organization-related outcomes: organizational commitment and employee productivity. An employee that is not engaged in work and instead spends time on digital leisure activities such as social media or watching a video is, perhaps needless to say, not working. Accordingly, for the most time, research on non-work-related digital activities has focused on the potential drawbacks of such activities on work performance [11]. However, this review found that digital leisure at work can be positively associated with productivity or performance outcomes. The current findings suggest that employee productivity would benefit from digital leisure by means of resource replenishment and recovery. Digital leisure activities in the workplace were related to, e.g., restoration of concentration [35], greater sustained task vigilance [24] as well as employee satisfaction [25] and, subsequently, predicted increased employee productivity. However, it should be noted that one reviewed study [35] found that the relationship between digital leisure and productivity is curvilinear and that time spent on digital leisure can be beneficial, but only up to an inflection point of 12 percent of working time.

Furthermore, recovery from work effort and demands can take place after work (external recovery) and during work (internal recovery) [15], for example by taking breaks from work. Findings of the reviewed literature suggest that, due to its easy and quick accessibility, digital leisure activities can function as a micro-break. Micro-breaks are short, informal breaks (i.e., not a ‘regular’ break) that last only a few minutes [41] and earlier research showed that micro-breaks can have an effect on—at least short-term—well-being at work [42]. Similar to recent findings on cyberloafing [43], these digital micro-breaks are, thus, potentially a ‘recovery tool’ for employees. While most studies in this review implicitly assumed that digital leisure activities can function as a (micro-)break, one study explicitly compared smartphone breaks to conventional breaks and found indeed evidence that smartphone breaks can improve employee energy levels and mental resilience [40]. In addition, Engaging in a corporate blog can enhance self-disclosure and improve common understanding among employees which, eventually, can increase affective organizational commitment [38].

### 4.1. Moderators and Mediators of Digital Leisure in the Workplace

Of the reviewed literature, only two articles investigated the possible role of moderators and mediators. This review found that age of a worker moderated the relationship of digital leisure and perceived employee productivity. This finding is perhaps not too unexpected. Leisure activities as a form of recovery are assumed to be self-chosen, pleasant activities [15]. As such, for workers that did not grow up in a world of constant digital connectivity, digital activities might not be construed as a source of leisure. However, as new workforces emerge that grew up with the Internet [7] and digital leisure, engaging in digital leisure at work is increasingly perceived as normal and, in a sense, expected. Furthermore, findings of one study suggest that employee satisfaction mediates the relationship between digital leisure and employee performance. In other words, digital leisure led to an increase in employee satisfaction which, subsequently, led to an increase in employee performance. This implies that as workers replenished mental resources and, thus, well-being, they were able to be more productive at work.

### 4.2. Future Research

Findings of this review show that digital leisure in the workplace can positively contribute to the workplace. Digital leisure activities can facilitate the recovery of mental resources and can improve employee productivity. As such, approaching non-work-related digital activities from a digital leisure perspective is important. Past research on such activities has heavily focused on the potential adverse effects and the employer’s perspective [11]. Therefore, a digital leisure perspective creates a more complete—and, hence, less biased— understanding of such digital activities. However, as has been pointed out, the field of leisure studies was slow to react to the relatively recent and sudden increase of digital leisure activities and sources [10] and, indicated by the small sample size of this review, research with an organizational focus is rare. Therefore, more research on digital leisure in the workplace is needed.

There are several avenues for future research extending the concept of digital leisure and its boundaries. As age might influence how a digital activity is construed (i.e., leisure or counterproductive work behavior), it is important to investigate the potential consequences among employees. For example, certain age groups might not reap the benefits of quickly accessible digital leisure activities as they do not actively engage in digital leisure. This might be especially true for the found effects of short-form videos on different facets of employee well-being that are dependent on video content (see Janicke-Bowles et al. [37]). Such effects (a sense of meaningfulness and relatedness at work) are not induced by other typical non-digital micro-break activities, such as looking out of the window [44]. Future research could develop practices to intentionally expose employees of all age groups to such short videos to ensure that the entire workforce can benefit.

Furthermore, potential conflict could arise between a new, younger workforce and their older colleagues as the generation not engaging in digital leisure might perceive their fellow coworkers as not being focused on the job or even less productive. Thus, future research should investigate the effects of employee attitudes towards digital leisure activities in the workplace among different age groups.

Additionally, this review found that the relationship between digital leisure and productivity was curvilinear. As such, investigating how employees manage and regulate their time spent on digital leisure and work might be a fruitful endeavor. This might be especially important for employees that work from home, where other regulatory factors (e.g., company policy) are absent or cannot be enforced.

Yet another variable of interest might be time spent on a digital leisure activity and its effects. Recent research on micro-breaks found evidence that shorter and more frequent breaks are more beneficial for resource recovery than fewer longer breaks [21], and one study in this review [35] found similar results for digital leisure in the form of general internet browsing and workplace productivity. Future research should investigate in more depth which digital activities, frequencies, durations, and possible combinations thereof are optimal to reap the benefits of digital micro-breaks.

Lastly, research should focus on organizational–contextual factors. As mentioned earlier, job autonomy is a necessity for leisure to take place [17]. Internal recovery (i.e., micro-breaks at work) can only be elicited if workers have control (autonomy) to actually be able to take such breaks [15] and strict internet monitoring can have adverse effects on work motivation and organizational commitment [45]. Therefore, investigating the potential moderating or mediating role of factors such as degree of job control and organizational leisure policies might be worthwhile.

In addition, based on the reviewed literature, methodological suggestions for future research can be made. As the majority of the included studies in this review used a cross-sectional design (surveys), future research should emphasize (quasi-)experimental research. While cross-sectional research certainly has its advantages (e.g., feasibility, large sample sizes), it cannot establish directionality or causality between the hypothesized associations [46]. In addition, a (quasi-)experimental or intervention design would allow to compare different sources, durations and frequencies of digital leisure and provide practical guidelines for policy making for the workplace.

Another suggestion concerns the measurement of workplace productivity. All of the reviewed cross-sectional studies used a measure of self-reported workplace productivity. Such measures are more prone to biases such as social desirability. Participants might have underreported to what extent they engage in digital leisure activities. Similarly, participants might overestimate or exaggerate their workplace productivity. Future research should, thus, strive to include additional sources of performance measures (reports), for example by including input from supervisors or peers.

### 4.3. Practical Implications

While the relatively small sample size of this review should be taken into account, the results of this review do have practical implications for the work context. Findings suggest that digital leisure activities in the workplace should not necessarily be considered counterproductive work behavior, but instead can function as a micro-break. These breaks can replenish exhausted mental resources and, as such, contribute to employee well-being and, perhaps more for younger workers, performance. Hence, organizational leisure policies should not entirely inhibit or prevent digital leisure and supervisors should make sure workers have a sufficient level of job autonomy to choose a digital leisure activity as recovery source. However, such activities might work best if they are not too long in duration. Instead, they should rather be no longer than four minutes, and more frequent. Establishing a corporate blog and encouraging employees to actively participate might improve socialization and identification with the organization.

### 4.4. Strengths and Limitations

The current review attempted to organize and synthesize the current state of knowledge regarding digital leisure activities in the workplace to gain a better understanding of how digital leisure contributes to positive outcomes in the workplace and under what circumstances. In order to do so, search terms were developed that evolved around digital leisure in the work context. As such, this review extended existing research on digital non-work-related activities in the workplace by including research findings from the field of leisure studies. Another strength of the included studies is that the studied population is culturally diverse (e.g., USA, Germany, China) that could decrease the risk of reproducing ethnocentric biases in scientific research.

However, as with any research, this review is not without its limitations. First, this review only collected a sample of eight citations. While several extensive searches were conducted within various databases, and papers were obtained by citation chaining, no attempts were made to collect additional papers by searching gray literature or contacting experts of the field. Second, as with any review, the period searched could be extended. While the field of digital leisure studies is relatively new and in an emerging state, searching more extensively, especially in combination with other search terms (e.g., ‘web’, ‘smartphone’), might yield different or more extensive results. The third limitation is that we did not calculate inter-rater reliability, although both authors met regularly to discuss the review and discrepancies. Documenting the inter-rater reliability is an important step to reduce subjectivity and bias of a systematic review [47], hence, the results of this review might be influenced by authors’ subjective interpretations. Fourth, positive publication bias can cause studies reporting non-significant associations regarding the effect of digital leisure engagement to remain unpublished [48]. In addition, given this review focuses on a specific life domain—individuals’ work life, caution is needed to generalize and apply the results of this review to other life domains and settings. Lastly, the present review only considers citations in the English language. While this might have introduced bias, it is not uncommon to limit searches to articles published in English [49]. However, considering the small sample size of included citations, future systematic reviews should extend the search parameters and explicitly search for articles published in other languages as well if the resources (e.g., language, translation costs, etc.) allow it.

## 5. Conclusions

The present paper systematically reviewed literature on digital leisure in the workplace, its positive outcomes and possible moderating and mediating variables. As digitization changes leisure activities and spaces, the current review challenges research that digital non-work-related activities are a counterproductive work behavior that should be prevented. Instead, findings suggest that, in moderation, digital leisure at work can contribute to employee overall well-being and productivity by means of mental recovery and replenishment. Understanding how and under which circumstance digital leisure in the workplace can have positive effects, can help organizations make the necessary changes to create a work environment in which workers and employers can reap the benefits of digital leisure engagement.

## Data Availability

Not applicable.
